# Dataset on passenger acceptance during autonomous ferry public trials: Questionnaires and interviews

**DOI:** 10.1016/j.dib.2024.110282

**Published:** 2024-03-02

**Authors:** Erik Veitch, Ole Andreas Alsos, Mina Saghafian, Felix-Marcel Petermann, Taufik Akbar Sitompul, Jooyoung Park

**Affiliations:** Department of Design, Norwegian University of Science and Technology (NTNU), Trondheim, Norway

**Keywords:** Questionnaire, Survey, Interview, Acceptance, Safety, Trust, Autonomous vehicles, Autonomous ferry, Qualitative

## Abstract

This paper presents a dataset from the first public trial of an urban autonomous passenger ferry. The dataset contains questionnaires designed to assess passenger acceptance in terms of perceived safety, trustworthiness, and reliability. Questionnaires and their responses are paired samples collected before and after use (N = 884). The dataset also contains transcripts of semi-structured interviews on the themes of perceived safety, trustworthiness, and reliability (N = 25). The public trial was held in Trondheim, Norway, during the period September-October 2022. The autonomous ferry used in the trial was the “milliAmpere2,” which is owned and operated by the Norwegian University of Science and Technology (NTNU). The data represents a state-of-the-art data collection effort owing to on-site data collection immediately before and after interactions with an Autonomous Vehicle (AV) in a public transportation context. The dataset is suitable for producing quantitative and qualitative analyses and for developing indicators of technology acceptance and related social phenomena regarding AVs, either in the maritime context or beyond.

Specifications Table

**Table:** 

Subject	Social Science, Safety Science
Specific subject area	Technology acceptance of Autonomous Vehicles (AVs) (defined as perceptions of safety, trustworthiness, and reliability).
Data format	Filtered data in both .csv format (comma separated file) and PDF/A format (transcripts of interviews).
Type of data	.csv file (dataset of questionnaires) .pdf file (transcripts of interviews)
Data collection	Questionnaires were collected on-site during public trials of the “milliAmpere2” autonomous ferry (*N* = 884). Questionnaires were derived from a variety of sources (cited in this article). Interviews were also conducted on-site (*N* = 25). This dataset spans 21 September to 2 October 2022. Copies of the questionnaires and interview protocols (English and Norwegian) are available in the data repository.
Data source location	Trondheim, Norway (63.434106 N, 10.392926S)
Data accessibility	Repository name: DataverseNO Data identification number: https://doi.org/10.18710/CFBQSN The archive is supported by the Norwegian University of Science and Technology (NTNU) and is hosted by UiT The Arctic University of Norway.

## 1 Value of the Data

• The dataset contains user feedback from the world's first public trial of an urban autonomous passenger ferry.
• These data are useful for understanding acceptance and attitudes of adults (18 years and over) towards AVs for public transportation.
• Dataset containing paired questionnaires collected before and after use (N = 884).
• Dataset containing interviews held immediately after use (N = 25).
• These data can be used in qualitative research about technology acceptance of autonomous ferries and of AVs in general.
• Researchers in social science as well as computer science, engineering, business, and design may benefit from this data.
• These data capture public AV use in a situated and social context, and as such go above and beyond conventional survey and simulation studies that do not capture first-hand interactions with the technology.
• These data are suitable for survey methods courses as well as for analyses conducted on the questionnaire and interview results.

## 2 Data Description

The “Before” and “After” questionnaires are available in the Norwegian University of Science and Technology DataverseNO repository as PDF files [1]. All respondents completed the questionnaires in-situ; that is, in person before or after using the autonomous ferry. The questionnaires are available both in English and Norwegian, as both were used in the data collection. Responses to both questionnaires are available in *questionnaire_results_r1*, which is a .csv file organized according to unique personal identifier (ID; see Table 1). The IDs may be useful for assigning labels to paired questionnaire (and interview) responses. The IDs do not disclose personal identities. The responses are labeled and coded accorded to the descriptions listed in Table 1.

**Table 1:** Questionnaire data column labels and descriptions of coded results (*N* = 884).

Label	Description
ID	Number used to identify passengers anonymously
Date	Date of data collection (dd/mm/yyyy)
Time	Approximate time of data collection (24 h)
Direction	0 = North crossing; 1 = South crossing (see Fig. 2)
Age	Age of passenger*
AQ1	1 = No; 0 = Yes
AQ2	0 = Commuting; 1 = Leisure; 2 = Curiosity; 3 = Other Reasons
AQ3-7	0 = Very Unsafe/Untrustworthy; 1 = Unsafe/Untrustworthy; 2 = Neither; 3 = Safe/Trustworthy; 4 = Very Safe/Trustworthy
BQ1-3	0 = Very Unsafe/Untrustworthy; 1 = Unsafe/Untrustworthy; 2 = Neither; 3 = Safe/Trustworthy; 4 = Very Safe/Trustworthy
BQ4	1 = No; 0 = Yes
BQ5	1 = No; 0 = Yes

⁎ Empty cells in the “Age” column indicate respondents who opted not to provide their age (105 empty cells). Note that this dataset has also been filtered to remove all passengers under the age of 18.

The only demographic information obtained from the questionnaires was age (i.e., gender, race/ethnicity, education, etc. was not collected). Only individuals 18 years and older are included in the dataset. Out of 884 total respondents to the questionnaires, 105 opted not to provide their age, leaving a total of *N* = 779 that provided age demographics. Table 2 lists the relevant age statistics.

**Table 2:** Descriptive statistics of age for participating respondents (*N* = 779).

Mean (SD)	44 (18)
Mode	24
Minimum	18
25th percentile	28
Median	42
75th percentile	57
Maximum	93

Age-related analyses on the dataset should be weighted relative to the population's age structure. Table 3 presents the age structure for the sample and for the population, which can be used to generate weights. The respondents’ age structure generally matched that of the population, except for the age group 20–29 years. This group is overrepresented by 10% relative to the general population and may introduce bias unless adjusted accordingly.

**Table 3:** Age structure for respondents and for population.

Age group	Respondents	Population*	Respondents (%)	Population (%)
0–9 years	0	22,169	0.0%	10.5%
10–19 years	8	23,144	1.0%	11.0%
20–29 years	223	38,718	28.6%	18.4%
30–39 years	136	32,482	17.5%	15.4%
40–49 years	112	26,744	14.4%	12.7%
50–59 years	128	25,049	16.4%	11.9%
60–69 years	84	19,855	10.8%	9.4%
70–79 years	73	15,000	9.4%	7.1%
80–89 years	13	5958	1.7%	2.8%
90–99 years	2	1345	0.3%	0.6%
100 years or older	0	32	0.0%	0.0%
*Total*	*779*	*210,496*		

*Population is for the municipality of Trondheim in 2022 [2].

The interview protocol is available in the DataverseNO repository (*Interview-guide-English.pdf* and *Intervjuguide-norsk.pdf*). Like for the questionnaires, both English and Norwegian protocols are included; however, only English transcripts are included. All files in the “Interview” folder are in PDF/A format. Filenames are labeled with date and ID according to the following format: *yyyy-mm-dd_ID_####.pdf*. One file has no date assigned (this is labeled with -*nd* in the *-mm-dd* field). Moreover, 11 files have no ID assigned (there are labeled with *_NA* in the _#### field). Some files have multiple IDs in the file name, indicating a group interview. The IDs can be used to match interview respondents with questionnaire respondents.

Transcriptions were completed by a third party and subsequently reviewed and edited by the interviewers for accuracy. The completed transcripts include verbalizations (e.g., “um”, “ah”) and include annotations in the form of question marks in instances where recordings were not comprehensible (e.g., “??”). Interviews lasted approximately 1–15 min (approximately 5 min, on average).

The original audio files are not included in the dataset to preserve participants’ anonymity.

## 3 Experimental Design, Materials and Methods

The method adopted a survey approach to data collection and leveraged in-situ sampling immediately before and after interacting with the AV. To design the “Before” and “After” questionnaires, questions were inspired by similar survey-based studies in peer-reviewed literature with a focus on acceptance of autonomous ferries [3,4]. To obtain feedback about safety, trustworthiness, and reliability, a five-point, self-reported Likert scale was chosen given its relevance for capturing information about difficult-to-observe phenomena [5]. The questionnaire was iterated and tested internally within a group of two researchers and five bachelor students for length, comprehensibility, and accuracy. Results of the pre-testing converged on the final version of the questionnaire.

Participants for the questionnaires were sampled by convenience during the trials. During the three-week trial (which lasted from 21 September to 9 October 2022), approximately 1500 individual passengers took the ferry. From these, more than half (N = 884) filled out the paired questionnaires. Participants were sampled during the first two weeks of the trial.

Interviews were held individually and in groups using a convenience sampling protocol of passengers disembarking the ferry. Most interview participants had also filled out the survey (14 out of 25 interviews contained one or more survey respondents), yet sampling was not exclusive to survey respondents. The interviews were semi-structured, in line with our qualitative case study approach [6]. No participants were rewarded in any way for their participation in the study.

**Fig. 1:**
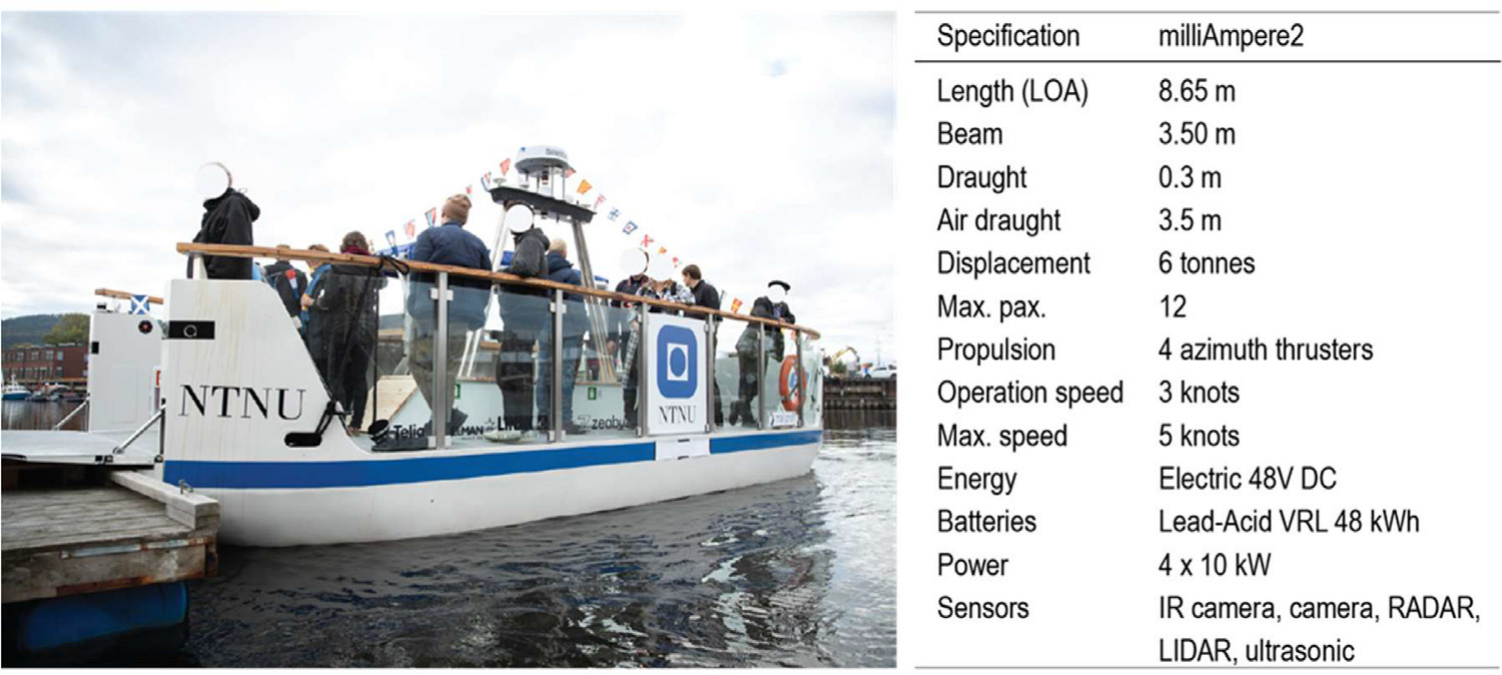
Main specifications of the “milliAmpere2” autonomous urban ferry.

The “milliAmpere2” ferry featured as the AV in the experimental design (Fig. 1). The “milliAmpere2” is an electric autonomous urban ferry owned and operated by NTNU. It is the advanced prototype version of the “milliAmpere,” which was used to develop the concept and its underlying computer and sensor architecture [7]. During the 2022 public trials, the ferry operated at a speed of approximately 3 knots and completed approximately 500 crossings in autonomous mode. A “safety host” was onboard during all crossings to comply with Norwegian regulations for passenger vessels under 12 m. The safety host had a maritime certificate (minimum “Class 5″; see [8]) as well as IMO50 certification for maritime safety training.

**Fig. 2:**
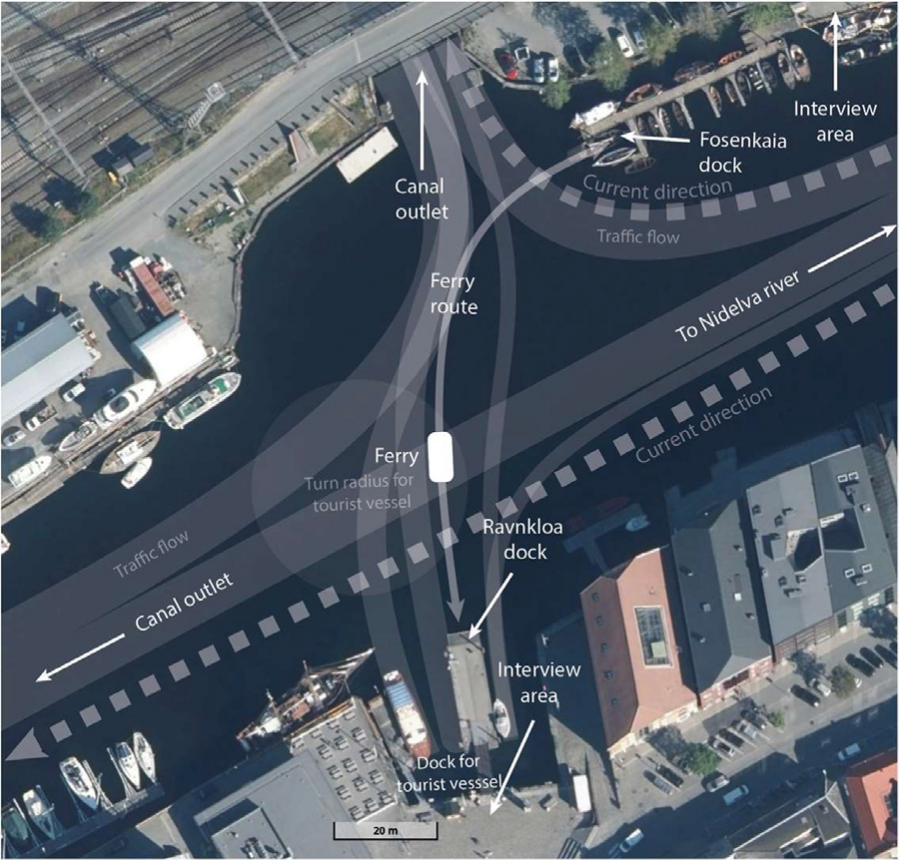
Characterization of the “milliAmpere2” ferry public trial location.

The location of the “milliAmpere2” crossing was a 100-m canal located in Trondheim, Norway (Fig. 2; 63.434106 N, 10.392926S). The crossing location traversed the equivalent of a three-way intersection and is well-trafficked by tourist boats, leisure boats, and other small craft. The speed limit in the canal is 5 knots. Note that references in the interview dataset to “Ravnkloa” and “Fosenkaia” indicate the South and North docks, respectively (Fig. 2).

## Limitations

This dataset includes only English transcriptions (*N* = 25). Interviews held in Norwegian are not included in the dataset. Overrepresentation of the age group 20–29 years in relation to the general population may introduce bias, which should be accounted for in any post-hoc analysis.

## Ethics Statement

This dataset involves human participants and contains some personal information (e.g., age). As such, compliance with ethical standards in research was of central importance to the experimental design. This study was audited and approved by the Norwegian Agency for Shared Services in Education and Research (Sikt), which oversees ethical conduct of research at Norwegian institutions. The data collection and management plan described herein were assigned the Sikt project number 340097.

The main measures in place to comply with Sikt's ethical research standards included informed consent and anonymity. Informed consent was obtained from all participants in the study (the consent form is included in the “Before” questionnaire, available in the dataset). Anonymity was maintained by assigning participant-independent numeric codes to participants and by removing potentially de-anonymizing text from interview data post-hoc.

## CRediT authorship contribution statement

**Erik Veitch:** Writing – original draft, Conceptualization, Investigation. **Ole Andreas Alsos:** Writing – review & editing, Conceptualization, Investigation, Supervision, Visualization. **Mina Saghafian:** Conceptualization, Methodology, Investigation. **Felix-Marcel Petermann:** Methodology, Investigation. **Taufik Akbar Sitompul:** Investigation. **Jooyoung Park:** Investigation.

## Data Availability

Questionnaire and interview data on passenger safety perception during autonomous ferry public trials (Original data) (Dataverse).
